# Synthesis of 1*H*-Pyrrolo[3,2-*g*]isoquinoline Derivatives as Ligands Targeting Haspin Kinase

**DOI:** 10.3390/molecules30224388

**Published:** 2025-11-13

**Authors:** Killian Malosse, Béatrice Josselin, Sandrine Ruchaud, Fabrice Anizon, Francis Giraud, Pascale Moreau

**Affiliations:** 1 ICCF, Clermont Auvergne INP, CNRS, Université Clermont Auvergne, F-63000 Clermont-Ferrand, France; killian.malosse@uca.fr (K.M.); fabrice.anizon@uca.fr (F.A.); 2 Station Biologique, Protein Phosphorylation and Human Diseases Unit, Plateforme de Criblage KISSf (Kinase Inhibitor Specialized Screening Facility), CNRS, Sorbonne Université, Place Georges Teissier, F-29688 Roscoff, France; 3 Station Biologique, CNRS UMR8227, Sorbonne Université, Place Georges Teissier, CS90074, F-29688 Roscoff cedex, France

**Keywords:** pyrroloisoquinolines, kinase inhibition, Haspin

## Abstract

A new series of 1*H*-pyrrolo[3,2-*g*]isoquinolines, diversely substituted at the 3-position either by a heteroaromatic scaffold or by propionate/acrylate side chains, were synthesized and evaluated as Haspin kinase inhibitors. The results of the kinase inhibitory potency study demonstrated that some of the new prepared compounds exhibited low nanomolar potencies toward Haspin. These results indicated that 3-substituted pyrrolo[3,2-*g*]isoquinolines could serve as intermediates for the development of PROTACs targeting Haspin, with the 3-position allowing further introduction of linkers to tether an E3 ligase ligand. However, this hypothesis remains to be demonstrated.

## 1. Introduction

Protein kinases represent promising biological targets for developing novel therapeutic strategies against cancer and other diseases, owing to their pivotal role in regulating cell signaling pathways [1]. These transferase enzymes catalyze the transfer of the γ-phosphate group of ATP to serine/threonine and/or tyrosine residues on protein substrates, thereby modulating their activation status. The discovery of Gleevec primarily targeting the Bcr-Abl kinase in the early 2000s provided the proof of concept that validated protein kinases as relevant therapeutic targets, especially in oncology [2]. Since then, extensive research efforts have been directed toward the identification and development of new protein kinase inhibitors, resulting in more than 80 small-molecule inhibitors approved by the FDA [3].

**Figure 1:**
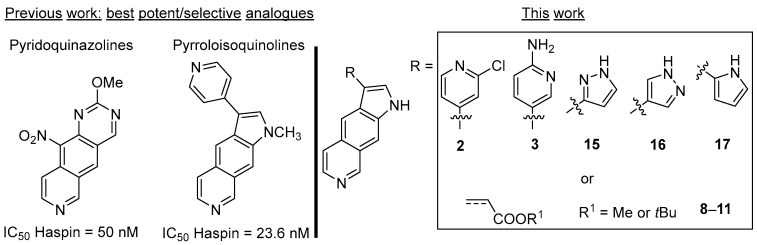
Structures of previously reported pyridoquinazoline and pyrroloisoquinoline derivatives exhibiting the most potent and selective inhibitory activity against Haspin kinase. Structures of newly synthesized pyrrolo[3,2-*g*]isoquinolines described in this work.

In the course of our research focused on designing and identifying novel heteroaromatic inhibitors of Haspin kinase, we recently reported new pyridoquinazolines [4] and pyrroloisoquinolines [5,6] demonstrating potent inhibitory activities in the low nanomolar range (Figure 1).

Due to its essential role in controlling mitosis via the regulation of Aurora proteins, Haspin (GSG2, Germ Cell-Specific Gene 2 Protein), an atypical serine/threonine kinase responsible for phosphorylating the Thr3 residue of Histone H3 in mitotic cells, represents a highly attractive therapeutic target in cancer treatment [7]. It has already been reported that Haspin is overexpressed in various cancer cell types in comparison to corresponding healthy ones [8,9]. For example, Haspin was found to be overexpressed in human lymphomas [10] and pancreatic [11] and bladder cancer tissues [12]. In addition, Haspin primarily exerts its functions in proliferating cells; thus, its inhibition should affect non-proliferating cells to a lesser extent, which should result in limited side effects [13].

**Figure 2:**
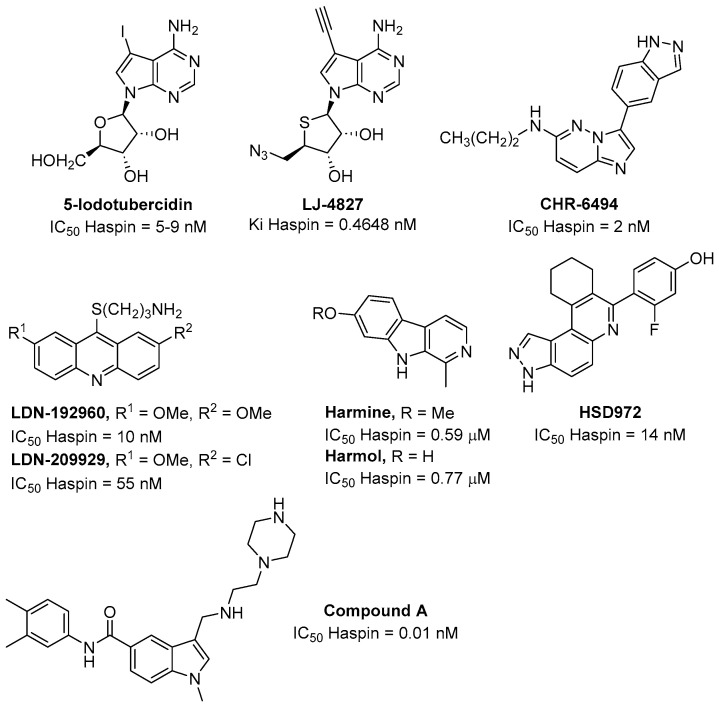
Examples of Haspin inhibitors from diverse chemical series reported by other groups.

Therefore, diverse classes of small-molecule inhibitors targeting Haspin, such as adenosine analogues (5-iodotubercidin [14], LJ-4827 [15]), imidazopyridazines (CHR-6494 [13]), acridines (LDN-192960 and LDN-209929 [16]), β-carbolines (harmine and harmol [17]), pyrazoloquinolines (HSD972 [18]), and indoles (compound **A** [19]), have been reported by multiple research groups (Figure 2).

**Figure 3:**
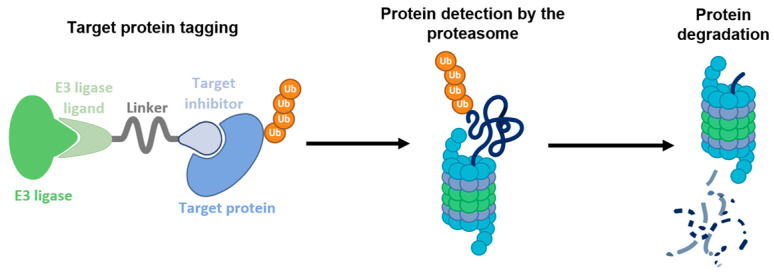
Schematic description of PROTAC strategy.

Currently, the PROTAC (PROteolysis Targeting Chimera) strategy is increasingly employed to modulate the level of specific proteins, including protein kinases. Unlike traditional inhibitors, PROTACs promote degradation of a protein of interest instead of simply inhibiting its activity. A PROTAC molecule typically comprises three components: a specific ligand of the targeted protein, an E3 ubiquitin ligase ligand, and a linker connecting these two entities. This design facilitates the formation of a ternary complex bringing the protein of interest into proximity with the E3 ligase. Consequently, the target protein undergoes polyubiquitination and subsequent degradation by the proteasome (Figure 3). Thus, PROTACs offer significant advantages over conventional small-molecule inhibitors, including greater potency at lower doses and prolonged effects even after the compounds have been cleared from the body [20,21].

Despite the rapidly growing number of studies focusing on developing novel PROTACs, as well as the identification of potent Haspin inhibitors across various chemical series, to our knowledge, no PROTAC specifically targeting Haspin has been reported to date. Therefore, to overcome this challenge and adapt this PROTAC strategy in this series, we have developed new pyrroloisoquinolines inhibiting Haspin kinase that could allow the attachment of a suitable linker connected to an E3 ligase ligand (Figure 1). This report describes the synthesis of new small molecules belonging to the 1*H*-pyrrolo[3,2-*g*]isoquinoline series as well as their kinase inhibitory potency against Haspin and other protein kinases known to be frequently cross-inhibited.

## 2. Synthesis and Biological Activity of New Pyrrolo[3,2-*g*]isoquinolines

**Scheme 1:**
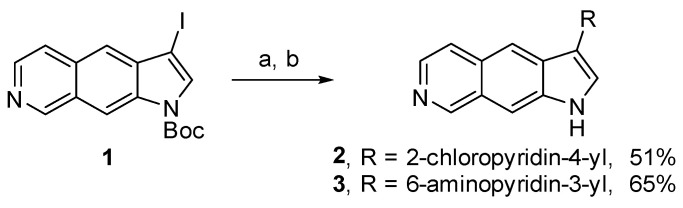
Synthesis of pyrroloisoquinolines **2** and **3**. Reagents and conditions: (a) 2-Chloropyridin-4-boronic acid (**2**), 6-Aminopyridine-3-boronic acid pinacol ester (**3**), Pd(PPh_3_)_4_, Na_2_CO_3_, DMF/H_2_O, microwave irradiation, 80 °C; (b) TFA, CH_2_Cl_2_.

In our previous studies, we demonstrated that potent Haspin inhibitory potency could be achieved within this series, with compounds substituted at the 3-position by halogen atoms or heteroaromatic moieties (e.g., pyridines [5,6]). Therefore, we first undertook the preparation of analogues **2**–**3** substituted at the 3-position by a functionalized pyridine ring which could be further used for linker attachment in a PROTAC approach (Scheme 1). Compounds **2** and **3** were prepared in 51% and 65% yields, respectively, in two steps from pyrroloisoquinoline **1** [6] by Suzuki cross-coupling under microwave irradiation and subsequent Boc cleavage using TFA in CH_2_Cl_2_ (Scheme 1). The first step used either commercial boronic acid (for the preparation of **2**) or synthesized pinacol boronate obtained in two steps from 2-aminopyridine by bromination then borylation (for the preparation of **3**) [22,23].

To extend the structure–activity relationship study around this scaffold, new pyrroloisoquinolines bearing a five-membered heterocyclic ring such as a pyrrole or pyrazole at the 3-position were prepared. These series would enable us to determine the influence of the ring size on the biological activity. Nitrogen atom of these rings could also be considered as linker attachement points, opening new outlooks for the design of promising PROTAC candidates.

**Scheme 2:**
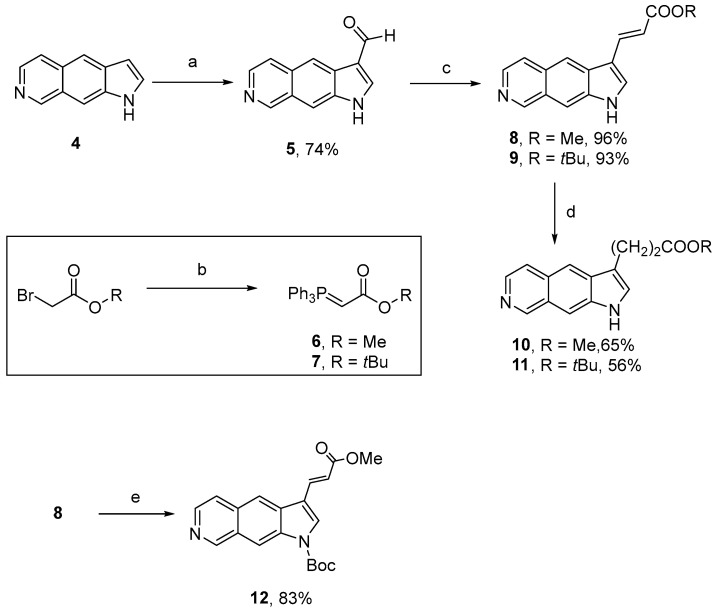
First synthetic route to pyrroloisoquinolines diversely substituted at the 3-position. Reagents and conditions: (a) HMTA, AcOH/H_2_O 1:2; (b) PPh_3_, AcOEt, 18 h; then, NaOH, H_2_O, CH_2_Cl_2_; (c) **6** or **7**, toluene; (d) H_2_, Pd(OH)_2_/C, MeOH; (e) Boc_2_O, DMAP, Et_3_N, CH_2_Cl_2_.

For this purpose, 3-formylated analogue **5** was first prepared as previously reported from indole **4** and hexamethylenetetramine in a Duff-type reaction (Scheme 2) [5]. Next, we prepared compounds **8**–**11**, which could be further used to afford pyrrole analogues. Thus, the reaction of compound **5** with freshly synthesized phosphorus ylides **6** or **7**, prepared from methylbromoacetate or *t*-butylbromoacetate [24], led to compounds **8** and **9** in 96% and 93% yields, respectively [25]. Catalytic hydrogenation of **8** and **9** double bonds also provided propionate analogues **10** and **11** (Scheme 2).

Construction of a pyrrole ring at the 3-position was then considered from compound **8**, which was treated with tosylmethylisocyanide (TosMIC) under basic conditions [26]. Unfortunately, ring formation did not occur, possibly due to the presence of an acidic proton on the heterocyclic scaffold. Therefore, **8** was Boc-protected to give **12**, before reaction with TosMIC under basic conditions using either NaH or *t*BuOK. Regrettably, none of these conditions led to the desired product (Scheme 2).

**Scheme 3:**
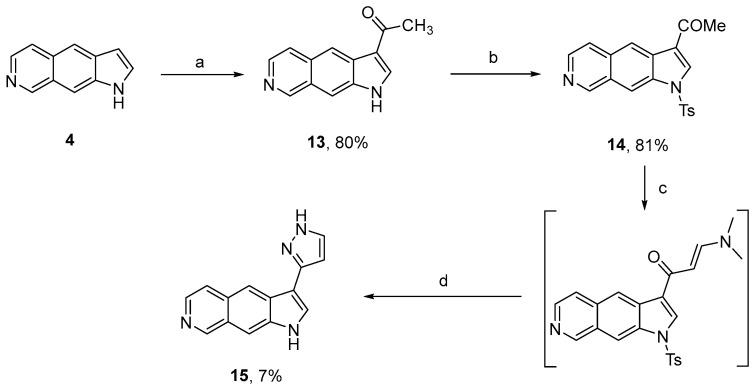
Synthetic route to pyrroloisoquinoline **15** bearing a pyrazole nucleus on position 3. Reagents and conditions: (a) AlCl_3_, CH_3_COCl, CH_2_Cl_2_; (b) DIPEA, DMAP, TsCl, CH_2_Cl_2_; (c) DMF-DTA, DMF; (d) H_2_N-NH_2_^.^HCl, K_2_CO_3_, 2-methoxyethanol.

In parallel, in order to prepare a pyrazole derivative, compound **4** was converted into analogue **14** after Friedel–Crafts acylation and protection of the indolic nitrogen atom by a tosyl group as previously described (Scheme 3) [5]. Reaction with dimethylformamide di-*tert*-butylacetal (DMF-DTA) led to an enaminone intermediate whose formation was confirmed by ^1^H-NMR analysis showing two doublets at 6.02 ppm and 7.69 ppm (*J* ~ 12.0 Hz). Condensation with hydrazine monohydrochloride in 2-methoxyethanol produced pyrazole analogue **15** in a poor yield [27].

**Scheme 4:**
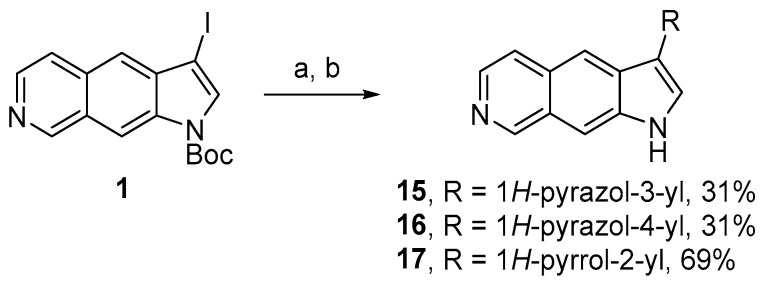
Synthesis of pyrroloisoquinolines **15**–**17**. Reagents and conditions: (a) pyrazole-3-boronic acid pinacol ester (**15**), pyrazole-4-boronic acid pinacol ester (**16**), *N*-Boc-pyrrole-2-boronic acid pinacol ester (**17**), Pd(PPh_3_)_4_, Na_2_CO_3_, DMF/H_2_O, microwave irradiation, 80 °C; (b) TFA, CH_2_Cl_2_.

These results indicated that these two synthetic approaches based on the formation of the 5-membered heterocyclic moiety (pyrrole or pyrazole) using 3-functionalized precursors were not effective. Therefore, as for the synthesis of pyridinyl derivatives **2** and **3**, we considered the cross-coupling strategy from **1** and either *N*-Boc pyrrole or pyrazole boronic pinacol esters. This approach successfully led to derivatives **15**–**17** bearing 5-membered heterocyclic rings at the 3-position in 31–69% yields (Scheme 4) [5].

We previouly reported that substitution at the 3-position with halogen atoms or pyridinyl or formyl groups was compatible with high potency toward Haspin kinase. To check if the current structural changes were in line with potent Haspin inhibition and high selectivity, enabling consideration of these compounds as possible targeting ligands for the PROTAC approach, compounds **2**, **3**, **8**–**11**, and **15**–**17** were evaluated toward a panel of eight protein kinases, including Haspin, as well as other kinases known to be frequently cross-inhibited (CLK1, DYRK1A, CDK9/Cyclin T, GSK-3β, CK1ε, CDK5/p25, and Pim1) (Table 1). The percentage of residual kinase activity was evaluated at 10 μM and 1 μM compound concentrations. Assays were performed in duplicate using the ADP-Glo assay [28] in the presence of 10 μM ATP. Haspin IC_50_ values were determined for compounds with Haspin residual kinase activity < 70% at a 1 μM compound concentration. In order to assess the selectivity profile of the best inhibitors, IC_50_ values were also measured for other kinases inhibited ≥50% at 1 μM.

Our results indicated that most potent Haspin inhibitors were heteroaromatic substituted derivatives **2** (bearing a 2-chloropyridin-4-yl substituent) and **3** (bearing a 6-aminopyridin-3-yl moiety) with IC_50_ values of 10.1 nM and 10.6 nM, respectively. For both compounds, two other protein kinases, DYRK1A and PIM1, were also inhibited, Haspin remaining the most impacted one. Regioisomers **15** and **16** bearing pyrazolyl subsitutents were also potent Haspin inhibitors with submicromolar potencies and good SI in regards to other protein kinases inhibited (SI > 7 in comparison to PIM1 or CLK1). Analogue **17** bearing a pyrrolyl moiety was a milder Haspin modulator with a submicromolar potency (IC_50_ value = 81.3 nM). However, compound **17** exhibited an excellent SI over CLK1, the only other kinase significantly inhibited in the panel evaluated. For other analogues 3-substituted with propionate or acrylate chains, the best results were obtained for methyl, rather than tert-butyl esters, with IC_50_ values of 15.5 nM and 82.1 nM for compounds **8** and **10**, respectively. Moreover, compound **8** was found to be selective for Haspin with an SI > 26 in regard to other protein kinases tested.

**Figure 4:**
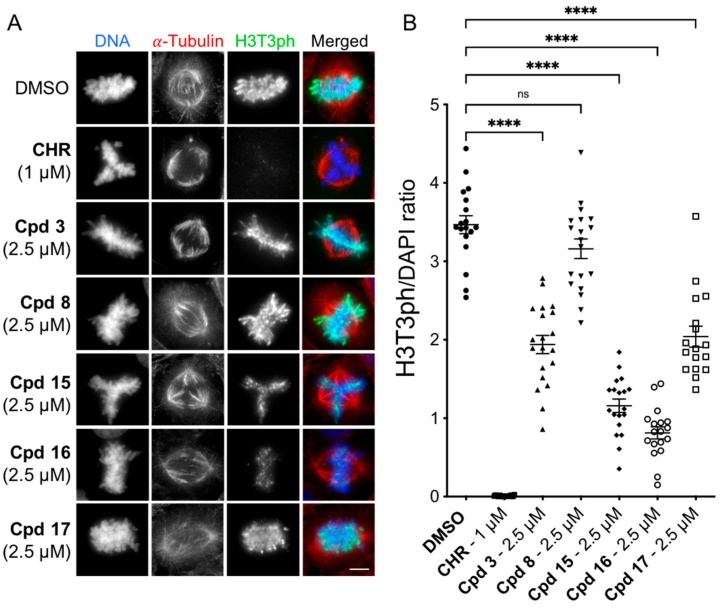
Evaluation of selected compounds on cellular endogenous Haspin activity. (**A**) U2OS cells were treated with 2.5 μM of each compound or 1 μM of CHR6494 (CHR) or DMSO for 16 h. Immunofluorescence assay was performed with anti-pH3T3 antibody (green) and anti-a-Tubulin (red); DNA was labelled with DAPI (blue). Representative images of early mitotic cells for each condition are presented. Scale bar: 5 μm. (**B**) Scatter plot representing H3T3ph/DAPI signal ratio indicative of Haspin activity; n > 17 mitotic cells per condition. n.s., not significant; **** *p* ≤ 0.0001.

In order to assess the ability of our most interesting compounds to inhibit cellular endogenous Haspin kinase, we evaluated their effects on the phosphorylation of Histone H3 on Thr3, a bona fide Haspin substrate (Figure 4). We treated osteosarcoma U2OS cells with 2.5 μM of selected compounds for 16 h before quantifying H3T3 phosphorylation by immunofluorescence on early mitotic cells (Figure 4A,B). The results showed that regioisomers **15** and **16** appeared as the most potent inhibitors, inhibiting endogenous Haspin activity by 66% and 77%, respectively, when compared to the DMSO control condition. Compounds **3** and **17** also significantly inhibited Haspin activity by around 43%. Unsurprisingly, the positive control, CHR6494, abolished Haspin activity. On the other hand, **8** did not show any significant inhibitory potential under these experimental conditions, despite an IC_50_ of 15.5 nM in vitro. This discrepency could be explained by the inability of this compound to cross the plasma membrane or by reduced stability in a cellular environment.

## 3. Conclusions

In summary, we synthesized novel 1*H*-pyrrolo[3,2-*g*]isoquinolines variously substituted at the 3-position with either heteroaromatic rings or propionate/acrylate side chains. The results of the kinase inhibitory potency study demonstrated that, in both cases, potent Haspin inhibition and selectivity could be achieved (compounds **3** and **15**–**17** in the heteroaromatic series—methyl esters **8** and **10** in the aliphatic series) with IC_50_ values of 10–80 nM. Additionaly, we showed that, except for **8**, all compounds were able to cross the cellular plasma membrane and inhibit endogenous Haspin kinase activity. Therefore, the best compounds identified in this work could be interesting intermediates to develop PROTACs targeting Haspin. That is, the substituents introduced at the 3-position of the pyrrolo[3,2-*g*]isoquinoline scaffold offer a handle for introducing a linker and an E3 ligase ligand, while conserving high binding affinity with Haspin. However, this hypothesis remains to be validated by additional research work that is currently ongoing in our laboratories.

## 4. Materials and Methods

### 4.1. Chemistry

#### 4.1.1. General

Starting materials were obtained from TCI (Tokyo, Japan), Acros Organics (Thermo Fisher Scientific, Geel, Belgium), Fluorochem (Hadfield, Derbyshire, UK), BLD Pharmatech Ltd. (Shanghai, China), and Alfa Aesar (Thermo Fisher Scientific, Ward Hill, MA, USA), and used without further purification. IR spectra were recorded on a Perkin-Elmer Spectrum 65 FT-IR spectrometer ($\bar{\nu }$ in cm^−1^) (PerkinElmer, Waltham, MA, USA). NMR spectra, recorded on a Bruker AVANCE 400 III HD (^1^H: 400 MHz, ^13^C: 101 MHz) (Bruker, Billerica, MA, USA), are reported in ppm using the solvent residual peak as an internal standard; the following abbreviations are used: singlet (s), doublet (d), doublet of doublets (dd), triplet (t), quadruplet (q), multiplet (m), broad signal (br s). Coupling constants are expressed in Hz. A CEM Discover 2.0 Benchmate apparatus (CEM, Matthews, NC, USA) was used for reactions performed under microwave irradiation in a sealed tube. A high-resolution Waters Micro Q-Tof or Thermo Scientific Q Exactive Q-Orbitrap apparatus (UCA START, Université Clermont Auvergne, Clermont-Ferrand, France) was used for high resolution mass spectrometric analysis. 40–63 μm silica gel was used for column chromatography purifications. Fluorescent silica gel plates (60 F254 from Macherey Nagel, Düren, Germany) were used for TLC monitoring. A Stuart SMP30 apparatus (Cole-Parmer, Vernon Hills, IL, USA) was used for melting point determinations. Melting points were uncorrected.

HPLC analyses using a VWR Hitachi chromatograph (Hitachi High-Tech Corporation, Tokyo, Japan) with DAD detector and a Macherey Nagel Nucleodur gravity column (4.6 mm × 250 mm, 5 µm) (Macherey-Nagel GmbH & Co. KG, Düren, Germany) were performed to determine the purity of all tested compounds. Analyses were performed at 25 °C, at 240 nm as detection wavelength and with a flow rate of 0.5 mL/min for each compound. Purity was determined by integration of chromatogram peaks (AUC). Solvent (A) was water containing 0.1% trifluoroacetic acid and solvent (B) was acetonitrile. The separation was achieved using a solvent gradient beginning with 95% (A) and 5% (B) for 5 min, followed by a linear increase to 5% (A) and 95% (B) over 25 min. 

^1^H and ^13^C NMR spectra of compounds **2**, **3**, **8**, **9**, **10**, **11** and **15**–**17** are provided as Supplementary Materials.

#### 4.1.2. General Synthetic Procedures

##### General Procedure A (Suzuki Coupling)

A 10 mL CEM Discover microwave tube was charged with compound **1** (1.0 eq.), the corresponding boronic acid or boronic ester (1.6 or 3.0 eq.), Na_2_CO_3_ (3.0 eq.), Pd(PPh_3_)_4_ (0.02 eq.) and a mix of DMF/H_2_O (4:1) (quantity necessary for a 75 mM final concentration) under argon. After tube sealing, the reaction mixture was irradiated for 2x15 min (Dynamic mode, Control Type = Standard, P = 175 W, T = 80–100 °C). Water was added to the mixture and EtOAc used for extraction. After washing with water and brine, drying over MgSO_4_, filtration and evaporation of the combined organic layers, the residue was solubilized in CH_2_Cl_2_/TFA (1:1) (50 mM final concentration). The solution was stirred 2 h at rt before solvent evaporation and addition of EtOAc and a saturated aqueous solution of Na_2_CO_3_. A column chromatography was used to purify the residue obtained after extraction twice with EtOAc, washing of the combined organic layers with brine, drying over MgSO_4_, filtration and evaporation under reduced pressure. 

##### General Procedure B (Phosphorus Ylide)

A solution of bromo derivative (1.0 eq.) in EtOAc (3.6 M concentration) was added to a stirred solution of PPh_3_ (1.05 eq) in EtOAc (0.6 M concentration). After 16 h, the white precipitate was collected by filtration, washed three times with Et_2_O, and dried. The solid was dissolved in CH_2_Cl_2_ (quantity necessary for a 0.5 M concentration), and NaOH (2.2 eq., aqueous solution at 1.3 M) was added. After 15 min of vigorous stirring, the phases were separated. The aqueous phase was washed with CH_2_Cl_2_, and the organic phases were combined, dried over MgSO_4_, filtered, and evaporated.

##### General Procedure C (Wittig)

To a screw-cap tube charged with **5** (1.0 eq.) and the corresponding ylide (1.3 eq.), toluene (quantity necessary for a 0.35 M final concentration) was added. The mixture was stirred overnight at 110 °C in an oil bath. After the completion of the reaction, the solution was concentrated under reduced pressure. The crude residue was purified by flash chromatography.

##### General Procedure D (Alkene Reduction)

A mixture of 20% Pd(OH)_2_/C (20% *w*/*w*) and the corresponding alkene in solution in MeOH (25 mM final concentration) was stirred under 1 atm. of H_2_ for 16 h at rt. The crude residue obtained after filtration through Celite, washing with MeOH and solvent evaporation was purified through column chromatography.

#### 4.1.3. Synthesis of Compounds **2**, **3**, **6**–**12**, and **15**–**17**

##### 3-(2-Chloro-pyridin-4-yl)-1*H*-pyrrolo[3,2-*g*]isoquinoline (**2**)

According to general procedure **A**, compound **2** was prepared from **1** (240 mg, 0.609 mmol) and 2-chloropyridine-4-boronic acid (153 mg, 0.974 mmol, 1.6 eq.). The reaction was performed at 100 °C. A column chromatography (CH_2_Cl_2_/MeOH 97:3 to 94:6) was performed to purify the mixture leading to **2** as a yellow powder (87.1 mg, 0.311 mmol, 51%). R*_f_* = 0.14 (EtOAc). Mp > 250 °C. IR (ATR): 3222-2588, 1589, 1438, 1255, 993 cm^−1^. ^1^H NMR (400 MHz, DMSO-*d*_6_): δ 7.94–7.96 (m, 3H), 8.23 (s, 1H), 8.30 (d, *J* = 5.6 Hz, 1H), 8.42 (dd, *J* = 4.4 Hz, *J* = 1.6 Hz, 1H), 8.66 (s, 1H), 8.67 (s, 1H), 9.41 (s, 1H), 12.29 (br s, 1H). ^13^C NMR (101 MHz, DMSO-*d*_6_): δ 108.5, 115.3, 119.6, 119.7, 120.5, 134.3, 139.0, 150.1, 152.7 (CH_arom_), 110.5, 125.0, 128.9, 129.8, 137.7, 146.2, 151.2 (C_arom_). HRMS (ESI^+^) *m*/*z* calcd for C_16_H_11_ClN_3_ (M + H)^+^ 280.0636, found 280.0632. HPLC: purity > 99%, t_R_ = 20.0 min.

##### 5-(1*H*-Pyrrolo[3,2-*g*]isoquinolin-3-yl)pyridin-2-amine (**3**)

According to general procedure **A**, compound **3** was prepared from **1** (76.1 mg, 0.193 mmol) and crude 6-aminopyridin-3-boronic acid pinacol ester (236 mg, 0.578 mmol, 3.0 eq.). The reaction was performed at 100 °C. The crude residue was purified by column chromatography (CH_2_Cl_2_/MeOH 96:4 to 92:8 + 1% Et_3_N) to give **3** as an orange powder (32.9 mg, 0.126 mmol, 65%). R*_f_* = 0.38 (CH_2_Cl_2_/MeOH 9:1). Mp > 80 °C (decomp.). IR (ATR): 3374-2507, 1600, 1498, 1436, 1247 cm^−1^. ^1^H NMR (400 MHz, DMSO-*d*_6_): δ 5.92 (s, 2H), 6.60 (d, *J* = 8.4 Hz, 1H), 7.81 (dd, *J* = 8.4 Hz, *J* = 2.4 Hz, 1H), 7.85 (d, *J* = 6.0 Hz, 1H), 8.01 (d, *J* = 2.4 Hz, 1H), 8.13 (s, 1H), 8.22 (d, *J* = 5.6 Hz, 1H), 8.35–8.37 (m, 2H), 9.35 (s, 1H), 11.64 (s, 1H). ^13^C NMR (101 MHz, DMSO-*d*_6_): δ 107.6, 108.3, 114.7, 120.5, 128.9, 136.1, 138.1, 145.3, 152.5 (CH_arom_), 112.6, 119.2, 124.8, 129.0, 130.2, 137.5, 158.0 (C_arom_). HRMS (ESI^+^) *m/z* calcd for C_16_H_14_N_4_ (M + 2H)^2+^ 131.0604, found 131.0603. HPLC: purity > 96%, t_R_ = 17.5 min.

##### Methyl (Triphenylphosphoranylidene)acetate (**6**)

According to general procedure **B** with methyl 2-bromoacetate (0.17 mL, 1.82 mmol) as starting material, after standard work-up, compound **6** was obtained as white powder (610 mg, 1.82 mmol, quant.). ^1^H NMR (400 MHz, DMSO-*d*_6_): δ 3.37 (s, 3H), 7.53–7.58 (m, 16H).

##### *tert*-Butyl (Triphenylphosphoranylidene)acetate (**7**)

According to general procedure **B** with *tert*-butyl 2-bromoacetate (0.54 mL, 3.63 mmol) as starting material, after standard work-up, compound **7** was obtained as white powder (1.29 g, 3.43 mmol, 94%). ^1^H NMR (400 MHz, DMSO-*d_6_*): δ 0.95 (56%) + 1.33 (44%) (s, 9H), 7.52–7.66 (m, 16H). *tert*-Butyl protons appeared as two singlets at 0.95 ppm and 1.33 ppm, probably due to the presence of geometrical isomers.

##### Methyl (*E*)-3-(1*H*-Pyrrolo[3,2-*g*]isoquinolin-3-yl)prop-2-enoate (**8**)

According to general procedure **C**, compound **8** was prepared from compounds **5** (175 mg, 0.892 mmol) and **6** (388 mg, 1.16 mmol). A column chromatography (CH_2_Cl_2_/MeOH 97:3 to 94:6) was performed to achieve purification of the mixture leading to **8** as a yellow powder (216 mg, 0.856 mmol, 96%). R*_f_* = 0.31 (CH_2_Cl_2_/MeOH 94:6). Mp > 198 °C (decomp.). IR (ATR): 3114-2420, 1711, 1622, 1601, 1427, 1148 cm^−1^. ^1^H NMR (400 MHz, DMSO-*d_6_*): δ 3.74 (s, 3H), 6.60 (d, *J* = 16.0 Hz, 1H), 7.96 (d, *J* = 6.0 Hz, 1H), 7.99 (d, *J* = 16.0 Hz, 1H), 8.19 (s, 1H), 8.31 (d, *J* = 6.0 Hz, 1H), 8.41 (d, *J* = 2.8 Hz, 1H), 8.57 (s, 1H), 9.40 (s, 1H), 12.21 (br s, 1H). ^13^C NMR (101 MHz, DMSO-*d_6_*): δ 51.1 (CH_3_), 111.2, 138.3 (CH_alkene_), 108.5, 116.3, 120.6, 138.8, 139.2, 152.7 (CH_arom_), 110.8, 125.2, 129.3, 130.1, 137.9 (C_arom_), 167.8 (C=O). HRMS (ESI^+^) *m/z* calcd for C_15_H_13_N_2_O_2_ (M + H)^+^ 253.0972, found 253.0968. HPLC: purity > 98%, t_R_ = 19.7 min.

##### *tert*-Butyl (*E*)-3-(1*H*-Pyrrolo[3,2-*g*]isoquinolin-3-yl)prop-2-enoate (**9**)

According to general procedure **C**, compound **9** was prepared from **5** (238 mg, 1.21 mmol) and compound **7** (594 mg, 1.58 mmol). A column chromatography (CH_2_Cl_2_/MeOH 97:3 to 96:4) was performed to achieve purification of the mixture. Then, the wax was solubilized in a minimum of CH_2_Cl_2_ before addition of Et_2_O to precipitate the product. After evaporation of solvents, **9** (332 mg, 1.13 mmol, 93%) was obtained as a yellow powder (332 mg, 1.13 mmol, 93%). R*_f_* = 0.40 (CH_2_Cl_2_/MeOH 94:6). Mp > 191 °C (decomp.). IR (ATR): 3144-2584, 1698, 1618, 1331, 1136 cm^−1^. ^1^H NMR (400 MHz, DMSO-*d_6_*): δ 1.52 (s, 9H), 6.48 (d, *J* = 15.6 Hz, 1H), 7.88 (d, *J* = 16.0 Hz, 1H), 7.98 (d, *J* = 5.6 Hz, 1H), 8.18 (s, 1H), 8.30 (d, *J* = 5.6 Hz, 1H), 8.37 (d, *J* = 2.4 Hz, 1H), 8.51 (s, 1H), 9.39 (s, 1H), 12.16 (br s, 1H). ^13^C NMR (101 MHz, DMSO-*d_6_*): δ 28.1 (3xCH_3_), 79.2 (C), 113.5, 137.3 (CH_alkene_), 108.4, 116.1, 120.6, 138.3, 139.2, 152.6 (CH_arom_), 110.8, 125.2, 129.4, 130.0, 137.9 (C_arom_), 166.8 (C=O). HRMS (ESI^+^) *m/z* calcd for C_18_H_19_N_2_O_2_ (M + H)^+^ 295.1441, found 295.1433. HPLC: purity > 97%, t_R_ = 22.0 min.

##### Methyl 3-(1*H*-Pyrrolo[3,2-*g*]isoquinolin-3-yl)propanoate (**10**)

According to general procedure **D**, compound **10** was prepared from **8** (102 mg, 0.404 mmol). The crude residue was purified by column chromatography (CH_2_Cl_2_/MeOH 97:3 to 96:4) to afford **10** as an orange powder (67.0 mg, 0.263 mmol, 65%). R*_f_* = 0.31 (CH_2_Cl_2_/MeOH 94:6). Mp = 126–127 °C. IR (ATR): 3161-2264, 1734, 1601, 1430, 1165 cm^−1^. ^1^H NMR (400 MHz, DMSO-*d_6_*): δ 2.78 (t, *J* = 7.6 Hz, 2H), 3.10 (t, *J* = 7.6 Hz, 2H), 3.60 (s, 3H), 7.60 (d, *J* = 2.0 Hz, 1H), 7.80 (d, *J* = 5.6 Hz, 1H), 8.05 (s, 1H), 8.12 (s, 1H), 8.20 (d, *J* = 5.6 Hz, 1H), 9.32 (s, 1H), 11.24 (br s, 1H). ^13^C NMR (101 MHz, DMSO-*d_6_*): δ 51.3 (CH_3_), 20.2, 33.9 (CH_2_), 107.0, 113.7, 120.2, 129.5, 138.1, 152.5 (CH_arom_), 112.5, 124.9, 128.3, 132.1, 137.2 (C_arom_), 173.0 (C=O). HRMS (ESI^+^) *m/z* calcd for C_15_H_15_N_2_O_2_ (M + H)^+^ 255.1128, found 255.1123. HPLC: purity > 99%, t_R_ = 19.8 min.

##### *tert*-Butyl 3-(1*H*-Pyrrolo[3,2-*g*]isoquinolin-3-yl)propanoate (**11**)

According to general procedure **D**, compound **11** was prepared from **9** (50 mg, 0.170 mmol). A column chromatography (CH_2_Cl_2_/MeOH 97:3) was performed to achieve purification of the crude residue. Then, the wax was solubilized in a minimum of CH_2_Cl_2_ before addition of Et_2_O to precipitate the product. After evaporation of solvents **11** (28.0 mg, 0.0945 mmol, 56%) was obtained as a yellow solid. R*_f_* = 0.42 (CH_2_Cl_2_/MeOH 94:6). Mp = 107–108 °C. IR (ATR): 3182-2623, 1718, 1601, 1429, 1122 cm^−1^. ^1^H NMR (400 MHz, DMSO-*d_6_*): δ 1.36 (s, 9H), 2.66 (t, *J* = 7.6 Hz, 2H), 3.05 (t, *J* = 7.6 Hz, 2H), 7.59 (d, *J* = 1.6 Hz, 1H), 7.80 (d, *J* = 5.6 Hz, 1H), 8.04 (s, 1H), 8.12 (s, 1H), 8.20 (d, *J* = 6.0 Hz, 1H), 9.32 (s, 1H), 11.22 (br s, 1H). ^13^C NMR (101 MHz, DMSO-*d_6_*): δ 27.7 (3xCH_3_), 20.4, 35.3 (CH_2_), 79.6 (C), 106.9, 113.7, 120.2, 129.4, 138.1, 152.5 (CH_arom_), 112.6, 124.9, 128.3, 132.1, 137.2 (C_arom_), 171.9 (C=O). HRMS (ESI^+^) *m/z* calcd for C_18_H_21_N_2_O_2_ (M + H)^+^ 297.1597, found 297.1594. HPLC: purity > 99%, t_R_ = 21.9 min.

##### *tert*-Butyl (*E*)-3-(3-Methoxy-3-oxoprop-1-en-1-yl)-1*H*-pyrrolo[3,2-*g*]isoquinoline-1-carboxylate (**12**)

To a solution of compound **8** (80 mg, 0.3171 mmol) in CH_2_Cl_2_ (2.1 mL) were added DMAP (3.9 mg, 0.0317 mmol), (Boc)_2_O (76 mg, 0.3488 mmol), and Et_3_N (66 µL, 0.4757 mmol). The mixture was stirred for 1 h at RT. Then the solution was diluted with water and extracted with CH_2_Cl_2_. The combined organic layers were washed with a saturated solution of NaCl, dried over MgSO_4_, filtered, and concentrated under reduced pressure. The residue was purified through column chromatography using CH_2_Cl_2_/MeOH (97:3) as eluant to give **12** (93 mg, 0.2639 mmol, 83%) as a very light-yellow solid. R*_f_* = 0.72 (CH_2_Cl_2_/MeOH 94:6). Mp = 151–152 °C. IR (ATR): 2981, 2939, 1708, 1631, 1453, 1346, 1229, 1144 cm^−1^. ^1^H NMR (400 MHz, DMSO-*d*_6_): δ 1.69 (s, 9H), 3.78 (s, 3H, CH_3_), 6.89 (d, 1H, *J* = 16.0 Hz), 7.99 (d, 1H, *J* = 16.4 Hz), 8.03 (d, 1H, *J* = 5.6 Hz), 8.46 (d, 1H, *J* = 6.0 Hz), 8.67 (s, 1H), 8.70 (s, 1H), 8.85 (s, 1H), 9.49 (s, 1H). ^13^C NMR (101 MHz, DMSO-*d_6_*): δ 27.7 (3xCH_3_), 51.4 (CH_3_), 85.0 (C), 117.2, 136.0 (CH_alkene_), 112.3, 117.6, 120.4, 135.7, 141.1, 153.2 (CH_arom_), 115.5, 126.1, 130.8, 131.7, 135.1 (C_arom_), 148.4, 167.0 (C=O). HRMS (ESI^+^): *m/z* calcd for C_20_H_21_N_2_O_4_ (M + H)^+^ 353.1496, found 353.1488.

##### 3-(1*H*-Pyrazol-3-yl)-1*H*-pyrrolo[3,2-*g*]isoquinoline (**15**)

To a solution of compound **14** (60 mg, 0.1646 mmol) in dry DMF (1.02 mL) under argon, dimethylformamide di-*tert-*butyl acetal was added (59 µL, 0.2470 mmol). The mixture was stirred for 6 h at 110 °C. A saturated aqueous solution of NaHCO_3_ was added and the aqueous layer was extracted with EtOAc. Combined organic layers were washed with water and brine, dried over MgSO_4_, and evaporated under reduced pressure. The crude enaminone was used in the next step without any further purification. To a solution of the enaminone in 2-methoxyethanol (0.7 mL) under argon were added hydrazine hydrochloride (17 mg, 0.2470 mmol) and K_2_CO_3_ (45 mg, 0.3292 mmol). The mixture was refluxed for 18 h. Then an aqueous saturated solution of Na_2_CO_3_ was added and the aqueous layer was extracted with EtOAc. The combined organic layers were washed with water and brine, dried over MgSO_4_, filtered, and evaporated under reduced pressure. The residue was purified by column chromatography (CH_2_Cl_2_/MeOH 95:5) to give **15** as a pale-yellow powder (2.64 mg, 0.0113 mmol, 7%).

According to general procedure **A**, compound **15** was alternatively prepared from **1** (80 mg, 0.203 mmol) and 1*H*-pyrazole-3-boronic acid pinacol ester (63 mg, 0.325 mmol, 1.6 eq.). The reaction was performed at 80 °C. The crude residue was purified by column chromatography (CH_2_Cl_2_/MeOH 93:7 to 91:9) to give **15** as an orange powder (14.6 mg, 0.0623 mmol, 31%).

R*_f_* = 0.15 (CH_2_Cl_2_/MeOH 92:8). Mp > 245 °C (decomp.). IR (ATR): 3224-2623, 1675, 1600, 1437, 1183 cm^−1^. Due to the presence of different tautomeric forms, some proton signals were poorly defined. The description provided corresponds to what was directly observable on the ^1^H spectrum. For the same reasons, two C_arom_ and two CH_arom_ signals were not detected in the ^13^C spectrum. ^1^H NMR (400 MHz, DMSO-*d*_6_): δ 6.74 (s, 1H), 7.74 (br s, 1H), 7.87 (d, *J* = 6.0 Hz, 1H), 8.14 (s, 1H), 8.21 (d, *J* = 2.4 Hz, 1H), 8.24 (d, *J* = 6.0 Hz, 1H), 8.68 (br s, 1H), 9.36 (s, 1H), 11.67 (br s, 1H), 12.67 (br s, 1H). ^13^C NMR (101 MHz, DMSO-*d*_6_): δ 101.6, 107.5, 120.5, 130.0, 138.4, 152.7 (CH_arom_), 125.0, 129.1, 129.7, 137.3 (C_arom_). HRMS (ESI^+^) *m/z* calcd for C_14_H_11_N_4_ (M + H)^+^ 235.0978, found 235.0975. HPLC: purity > 97%, t_R_ = 18.3 min.

##### 3-(1*H*-Pyrazol-4-yl)-1*H*-pyrrolo[3,2-*g*]isoquinoline (**16**)

According to general procedure **A**, compound **16** was prepared from **1** (120 mg, 0.304 mmol) and pyrazole-4-boronic acid pinacol ester (177 mg, 0.913 mmol, 3.0 eq.). The reaction was performed at 80 °C. A column chromatography (CH_2_Cl_2_/MeOH 95:5 to 9:1) was performed to achieve purification of the crude leading to **16** as an orange powder (22.6 mg, 0.096 mmol, 31%). R*_f_* = 0.23 (CH_2_Cl_2_/MeOH 92:8). Mp > 154 °C (decomp.). IR (ATR): 3340-2553, 1600, 1433, 1274, 1091 cm^−1^. ^1^H NMR (400 MHz, DMSO-*d*_6_): δ 7.86 (d, *J* = 6.0 Hz, 1H), 8.05 (d, *J* = 1.6 Hz, 1H), 8.10–8.17 (m, 3H), 8.23 (d, *J* = 5.6 Hz, 1H), 8.43 (s, 1H), 9.35 (s, 1H), 11.53 (s, 1H), 12.88 (br s, 1H). ^13^C NMR (101 MHz, DMSO-*d*_6_): δ 107.3, 115.0, 120.5, 128.6, 138.1, 152.5 (CH_arom_), 107.2, 114.3, 124.9, 128.9, 130.3, 137.3 (C_arom_). Due to tautomers, two CH_arom_ signals were not observed. HRMS (ESI^+^) *m/z* calcd for C_14_H_11_N_4_ (M + H)^+^ 235.0978, found 235.0975. HPLC: purity > 97%, t_R_ = 18.3 min.

##### 3-(1*H*-Pyrrol-2-yl)-1*H*-pyrrolo[3,2-*g*]isoquinoline (**17**)

According to general procedure **A**, compound **17** was prepared from **1** (80 mg, 0.203 mmol) and 1-Boc-pyrrole-2-boronic acid pinacol ester (95.3 mg, 0.325 mmol, 1.6 eq.). The reaction was performed at 80 °C. A column chromatography (CH_2_Cl_2_/MeOH 96:4) was performed to achieve the purification of the crude leading to **17** as a brown powder (32.6 mg, 0.140 mmol, 69%). R*_f_* = 0.18 (CH_2_Cl_2_/MeOH 94:6). Mp > 153 °C (decomp.). IR (ATR): 3324-2224, 1599, 1442, 1428, 1252, 1109 cm^−1^. ^1^H NMR (400 MHz, DMSO-*d*_6_): δ 6.18 (app. q, *J* = 2.8 Hz, 1H), 6.55–6.59 (m, 1H), 6.81–6.84 (m, 1H), 7.88 (d, *J* = 6.0 Hz, 1H), 8.02 (d, *J* = 2.4 Hz, 1H), 8.12 (s, 1H), 8.23 (d, *J* = 6.0 Hz, 1H), 8.45 (s, 1H), 9.36 (s, 1H), 11.08 (br s, 1H), 11.53 (br s, 1H). ^13^C NMR (101 MHz, DMSO-*d*_6_): δ 104.5, 107.3, 108.4, 115.3, 117.0, 120.5, 127.5, 138.3, 152.5 (CH_arom_), 108.8, 124.9, 125.9, 128.9, 129.5, 137.2 (C_arom_). HRMS (ESI^+^) *m/z* calcd for C_15_H_12_N_3_ (M + H)^+^ 234.1025, found 234.1022. HPLC: purity > 99%, t_R_ = 20.3 min.

### 4.2. In Vitro Kinase Inhibition Assays

Kinase inhibition was tested in 384-well plates using the ADP-GloTM assay kit (Promega, Madison, WI, USA) according to the procedure recommended by the manufacturer [28]. Reactions were set up in a final volume of 6 μL in an appropriate kinase buffer (10 mM MgCl_2_, 1 mM EGTA, 1 mM DTT, 25 mM Tris-HCl pH 7.5, 50 μg/mL heparin) with either protein or peptide as the substrate in the presence of 10 μM ATP. To stop the kinase reaction, 6 μL of ADP-GloTM Kinase Reagent was added after 30 min at 30 °C. Then, after 50 min of incubation at room temperature (RT), 12 μL of Kinase Detection Reagent was added. The transmitted signal was measured after 1 h at RT using the Envision (Perkin Elmer, Waltham, MA, USA) microplate luminometer and expressed in relative light units (RLU). In order to determine the half-maximal inhibitory concentration (IC_50_), all assays were performed in duplicate in the absence or presence of increasing doses of the tested compounds. GraphPad Prism10 software (GraphPad Software, San Diego, CA, USA) was used to fit dose–response curves and to determine the IC_50_ values. Kinase activities are given as percentages of maximal activity, i.e., measured in the absence of inhibitor. Peptide substrates were obtained from Proteogenix (Schiltigheim, France).

The following protein kinases were analyzed in this study: CDK5/p25 (human, vector pGEX-2T, expressed in bacteria; kindly provided by Dr. J. H. Wang) was assayed with 0.8 μg/μL of histone H1 as substrate; HsCDK9/CyclinT (NP_001252/KAI4065659, cloned in pTEFb vector, expressed by baculovirus in Sf9 insect cells) were assayed with 0.27 μg/μL of the following peptide: YSPTSPSYSPTSPSYSPTSPSKKKK, as substrate; HsCK1ε (NP_001885, cloned in pFAST-BAC vector, expressed in Sf9 insect cells) was assayed with 0.022 μg/μL of the following peptide: RRKHAAIGSpAYSITA (“Sp” stands for phosphorylated serine) as CK1-specific substrate; HsGSK3β (AAH12760, cloned in pFAST-BAC, expressed by baculovirus in Sf9 insect cells) were assayed with 0.010 μg/μL of GS-1 peptide, a GSK-3-selective substrate (YRRAAVPPSPSLSRHSSPHQSpEDEEE); HsHaspin-kd (NP_114171, kinase domain, amino acids 470–798 cloned in pGEX-6P-3, expressed in bacteria) was assayed with 0.007 μg/μL of histone H3 (1–21) peptide (ARTKQTARKSTGGKAPRKQLA) as substrate; Pim-1 (KAI4018123, expressed in bacteria) was assayed with 0.8 μg/μL of histone H1 as substrate; HsCLK1 (NP_004062, cloned in pFAST-BAC, expressed in Sf9 insect cells) was assayed with 0.027 μg/μL of the peptide: GRSRSRSRSRSR; RnDYRK1A-kd (*Rattus norvegicus*, amino acids 1–499, pGEX-2TK, expressed in bacteria, gift from Dr. W. Becker, Aachen, Germany) was tested in the presence of 0.033 μg/μL of the peptide: KKISGRLSPIMTEQ.

### 4.3. Cellular Evaluation: H3T3ph Immunofluorescence Assay

Ethical approval for this study was obtained (9 July 2025) from DGRI-LabOGM (APPROVAL NUMBER: L1-2373). U2OS cells (purchased from ATCC, Manassas, VA, USA**,** reference HTB-96) were grown in DMEM medium (Dulbecco’s Modified Eagle Medium, High glucose; GlutaMAX, Life Technologies, Carlsbad, CA, USA) supplemented with 10% fetal bovine serum (Gibco, Grand Island, NY, USA) in a 5% CO_2_ humidified atmosphere. Cells grown on coverslips were treated for 16 h with each compound or 0.025% DMSO, then fixed with 4% paraformaldehyde in PBS, permeabilized by 0.15% Triton-X100 for 2 min, and blocked for 12 min in 4% BSA in PBS. Immunofluorescence assay was performed using standard protocols with a rabbit anti-phospho-Thr3 Histone H3 and a mouse anti-a-Tubulin (B-5-1-2) obtained from Merck Millipore, Burlington, MA, USA (1/2000 dilution). Coverslips were mounted with Vectashield containing DAPI (Vector Laboratories, Newark, CA, USA). Images were acquired with a Coolsnap HQ_2_ CCD camera (Photometrics, Tucson, AZ, USA) on a Zeiss Axio microscope (Carl Zeiss, Oberkochen, Germany) using a 63x NA 1.46 objective. Image acquisition and processing were performed using Zen Blue (Carl Zeiss). The H3T3ph signal on chromosomes was normalized against the DAPI signal and analyzed using ImageJ software (NIH, Version 1.54p). Graphs were produced and statistical analysis was performed using GraphPad Prism 10. *p*-values were determined by *t*-test; n.s., not significant; **** *p* ≤ 0.0001.

## Figures and Tables

**Table 1:** Percentage of kinase residual activity at 10 μM and 1 μM compound concentrations (**2**, **3**, **8**–**11**, and **15**–**17**). IC_50_ values in nM (given in parentheses) were determined toward Haspin when residual activity was <70% at 1 μM. For other kinases, IC_50_ values were measured for most inhibited ones (% Inhibition ≥ 50% at 1 μM). Assays performed in duplicate. Typically, the standard deviation of single data points was <10%. SI: selectivity index = IC_50_ second most inhibited kinase/IC_50_ Haspin. ND: not determined (for these analyses, a problem in fitting the dose–response curve did not allow a reliable IC_50_ value determination). NE: not evaluated.

Cpds	Kinase Inhibition (% Residual Activity at 10 μM or 1 μM (IC_50_ Values))																
	Haspin		CLK1		DYRK1A		CDK9		GSK3		CK1		CDK5		PIM1		SI
	10	1	10	1	10	1	10	1	10	1	10	1	10	1	10	1	
**2**	8	9	27	56	16	20	56	81	81	76	54	79	100	100	14	20	
	(10.1)				ND										ND		
**3**	1	7	18	59	0	15	44	83	58	80	63	78	100	100	7	17	8.8
	(10.6)				(212.5)										(93.7)		(PIM1)
**8**	6	11	22	55	16	43	51	79	71	78	77	90	100	72	8	26	26.1
	(15.5)				(1128.0)										(404.4)		(PIM1)
**9**	12	30	48	86	28	51	32	79	80	80	87	92	100	100	21	42	
	(391.8)														ND		
**10**	10	45	60	91	32	72	72	90	99	100	97	100	100	100	52	72	
	(82.1)																
**11**	52	70	81	98	31	58	86	100	94	83	81	98	100	100	60	70	
**15**	0	0	4	32	1	39	NE	NE	86	100	48	100	76	98	0	44	7.4
	(30.6)		(503.2)		(323.8)										(221.9)		(PIM1)
**16**	0	0	0	4	0	11	NE	NE	17	94	12	77	10	91	0	20	8.2
	(12.4)		(102.0)		(163.4)										(1743)		(CLK1)
**17**	0	6	36	47	19	69	39	76	65	71	81	92	81	75	33	91	31.2
	(81.3)		(2522)														(CLK1)

## Data Availability

Data available upon request.

## Supplementary Materials

The following supporting information can be downloaded at https://www.mdpi.com/article/10.3390/molecules30224388/s1, ^1^H and ^13^C NMR spectra of compounds **2**, **3**, **8**, **9**, **10**, **11** and **15**–**17** are provided as supplementary materials.
