# Supplementary material for: Synthesis of 1H-Pyrrolo[3,2-g]isoquinoline Derivatives as Ligands Targeting Haspin Kinase
Source: Molecules. 2025 Nov 13;30(22):4388. doi: 10.3390/molecules30224388 (PMC12655252; doi:10.3390/molecules30224388)

Supplementary material

Synthesis of 1*H*-pyrrolo[3,2-*g*]isoquinoline derivatives as ligands targeting Haspin kinase

Killian Malosse[‡], Béatrice Josselin[†,§], Sandrine Ruchaud[§], Fabrice Anizon[‡], Francis Giraud[‡,*], Pascale Moreau[‡,*]

‡ Université Clermont Auvergne, CNRS, Clermont Auvergne INP, ICCF, F-63000 Clermont-Ferrand, France.

† Sorbonne Université, CNRS, Plateforme de Criblage KISSf (Kinase Inhibitor Specialized Screening Facility), Protein Phosphorylation and Human Diseases Unit, Station Biologique, Place Georges Teissier, F-29688 Roscoff, France.

§ Sorbonne Université / CNRS UMR8227, Station Biologique, Place Georges Teissier, CS90074, F-29688 Roscoff cedex, France.

* Correspondence: pascale.moreau@uca.fr (PM); francis.giraud@uca.fr (FG)

Table of contents:

^{1}H NMR and ^{13}C NMR spectra of compound **2** page 2
^{1}H NMR and ^{13}C NMR spectra of compound **3** page 3
^{1}H NMR and ^{13}C NMR spectra of compound **8** page 4
^{1}H NMR and ^{13}C NMR spectra of compound **9** page 5
^{1}H NMR and ^{13}C NMR spectra of compound **10** page 6
^{1}H NMR and ^{13}C NMR spectra of compound **11** page 7
^{1}H NMR and ^{13}C NMR spectra of compound **15** page 8
^{1}H NMR and ^{13}C NMR spectra of compound **16** page 9
^{1}H NMR and ^{13}C NMR spectra of compound **17** page 10

^{1}H NMR and ^{13}C NMR spectra of compound **2**

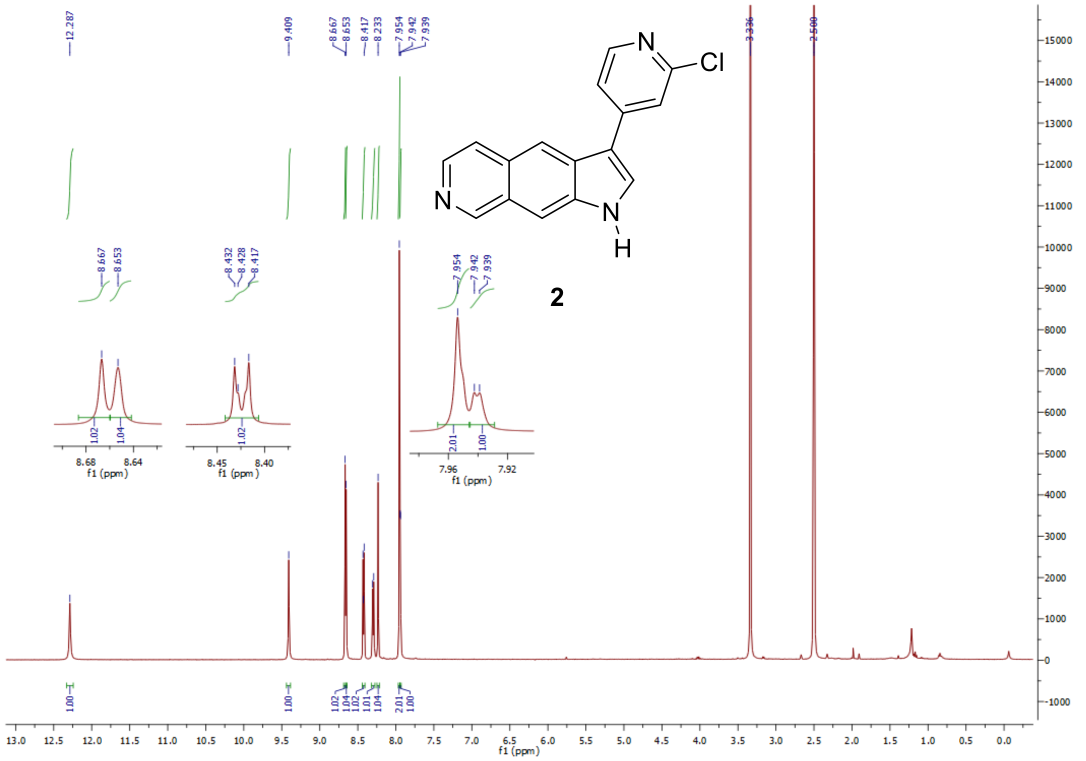

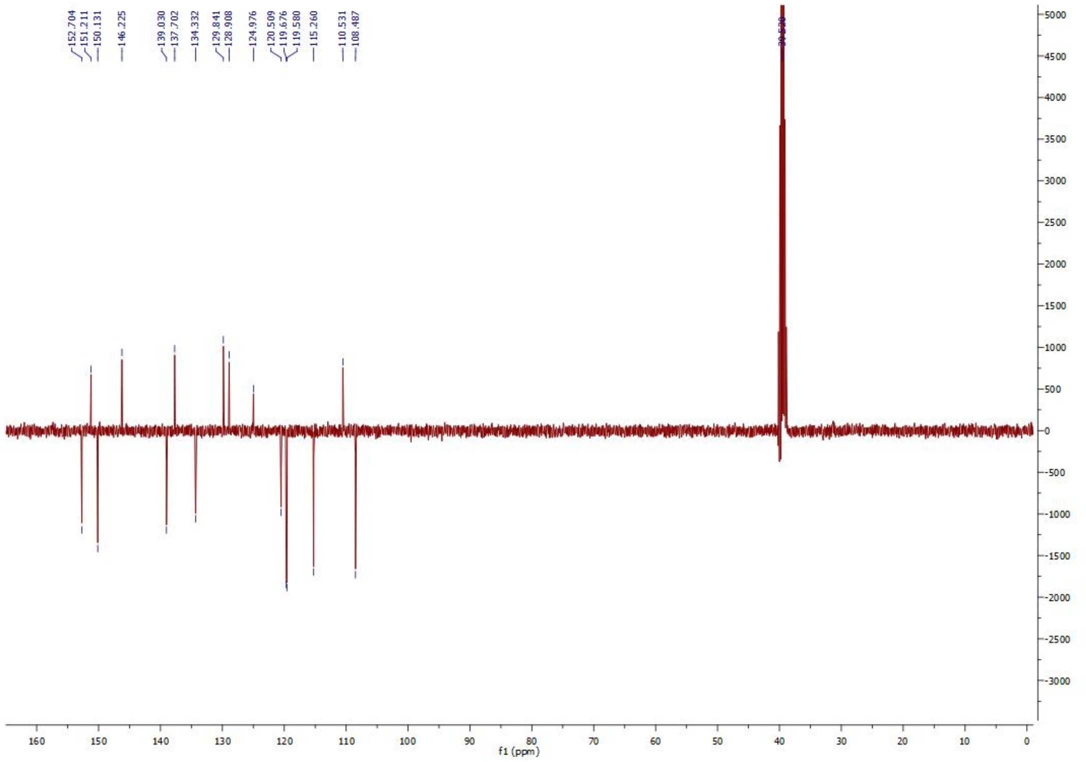

^{1}H NMR and ^{13}C NMR spectra of compound **3**

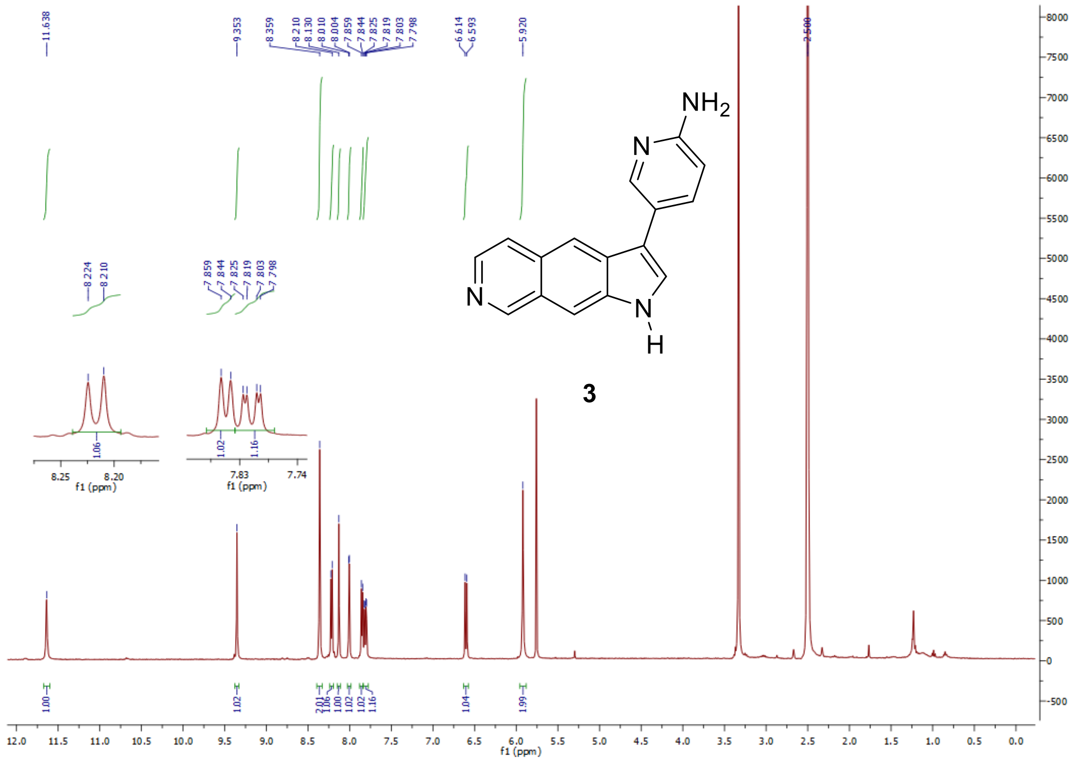

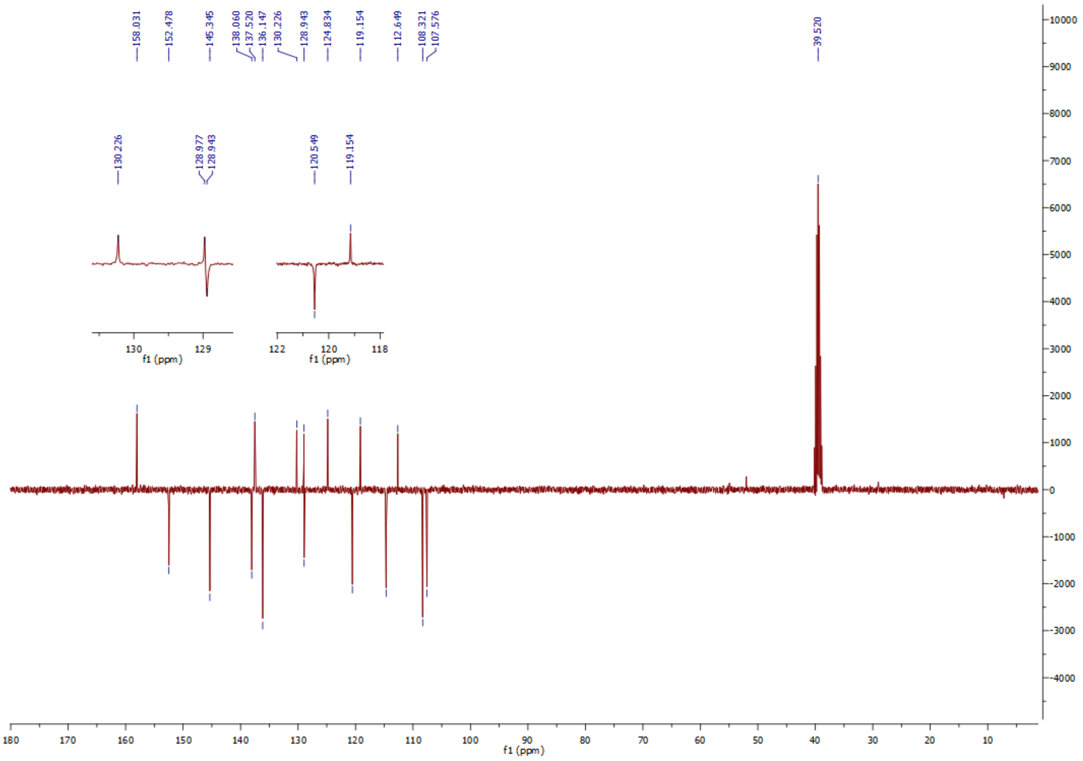

^{1}H NMR and ^{13}C NMR spectra of compound **8**

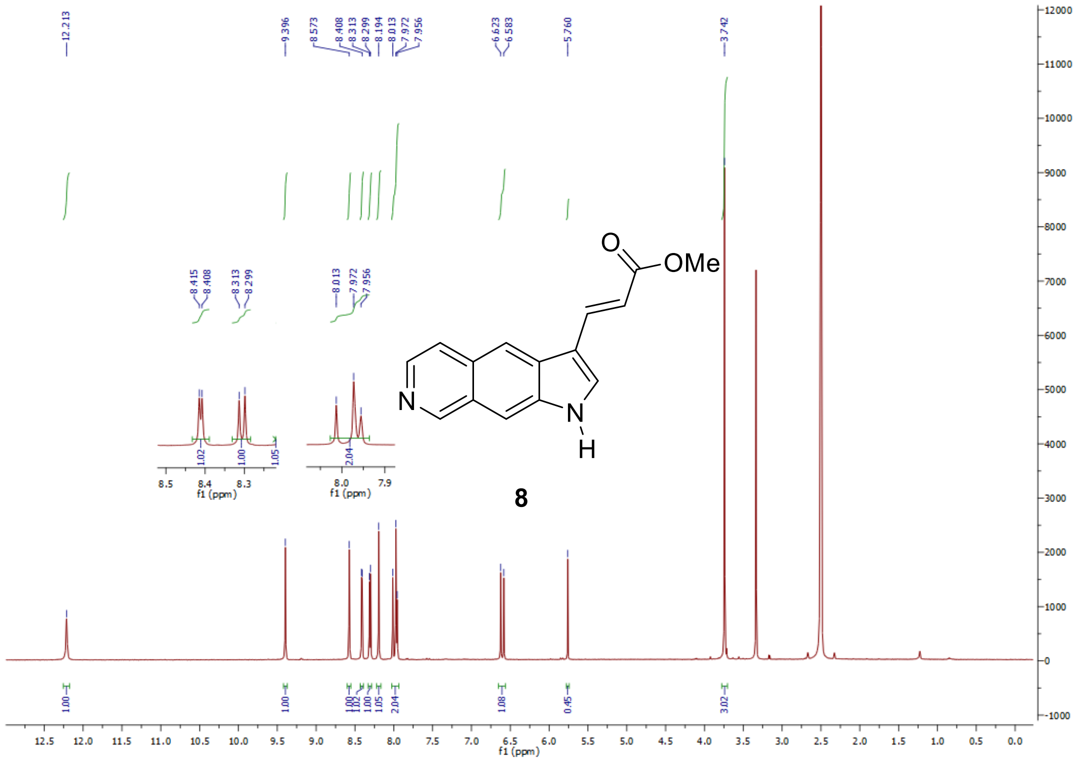

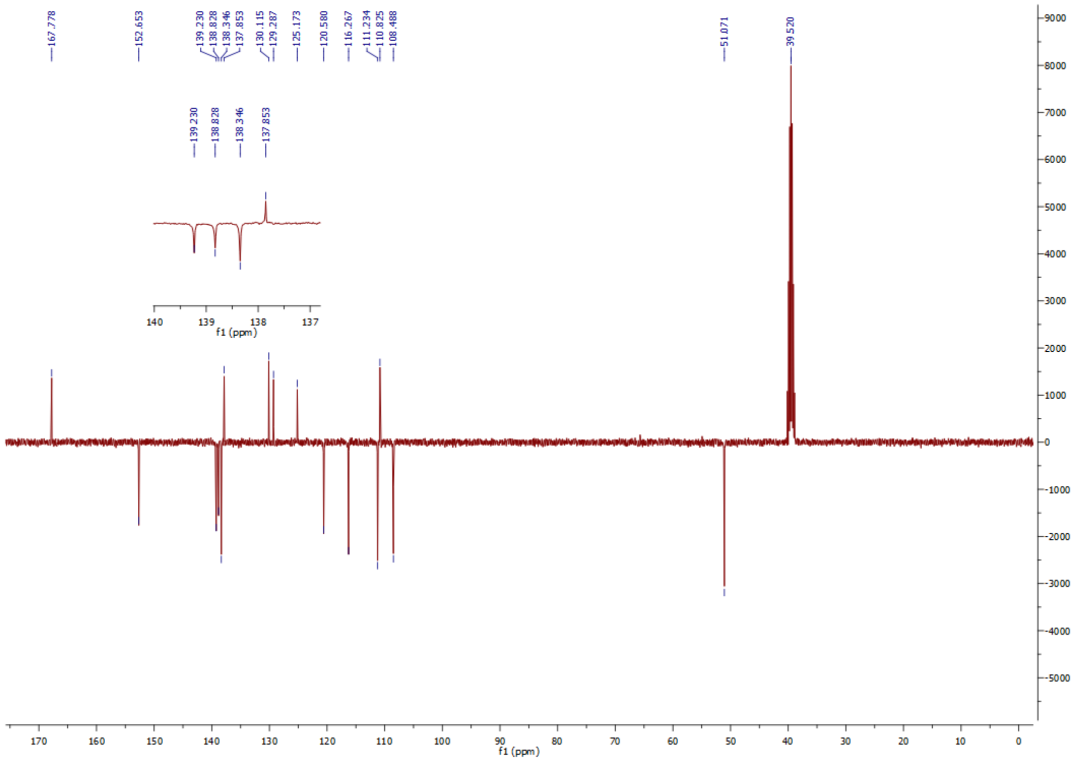

^{1}H NMR and ^{13}C NMR spectra of compound **9**

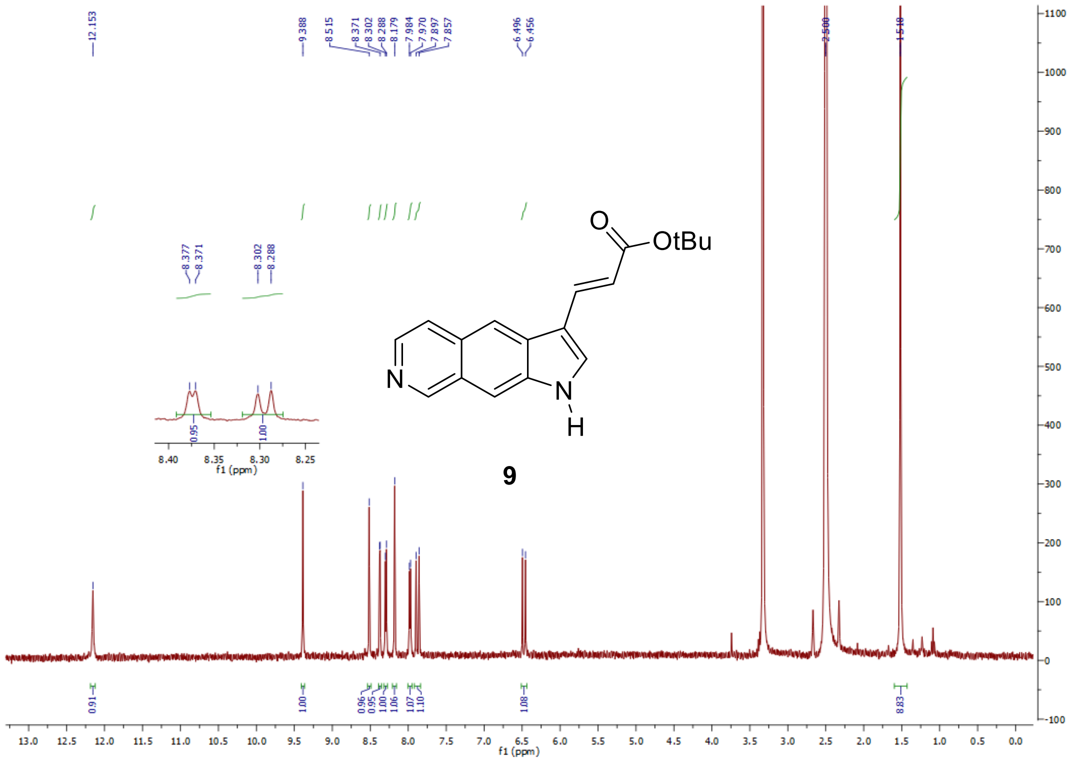

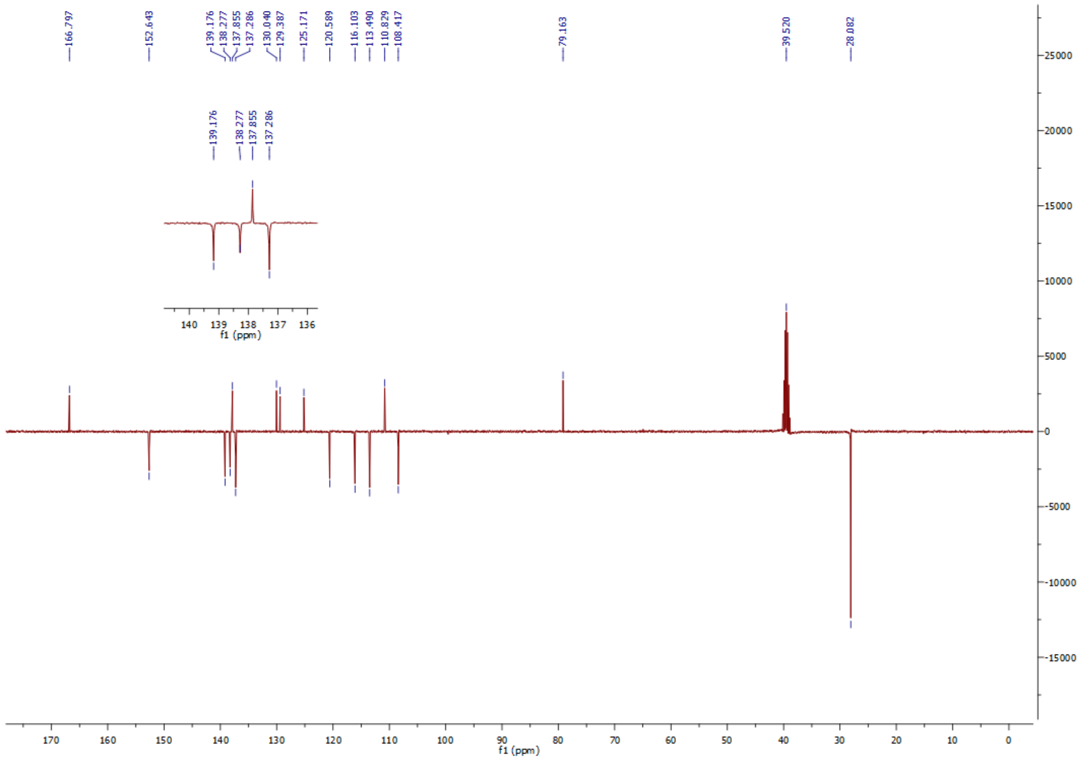

^{1}H NMR and ^{13}C NMR spectra of compound **10**

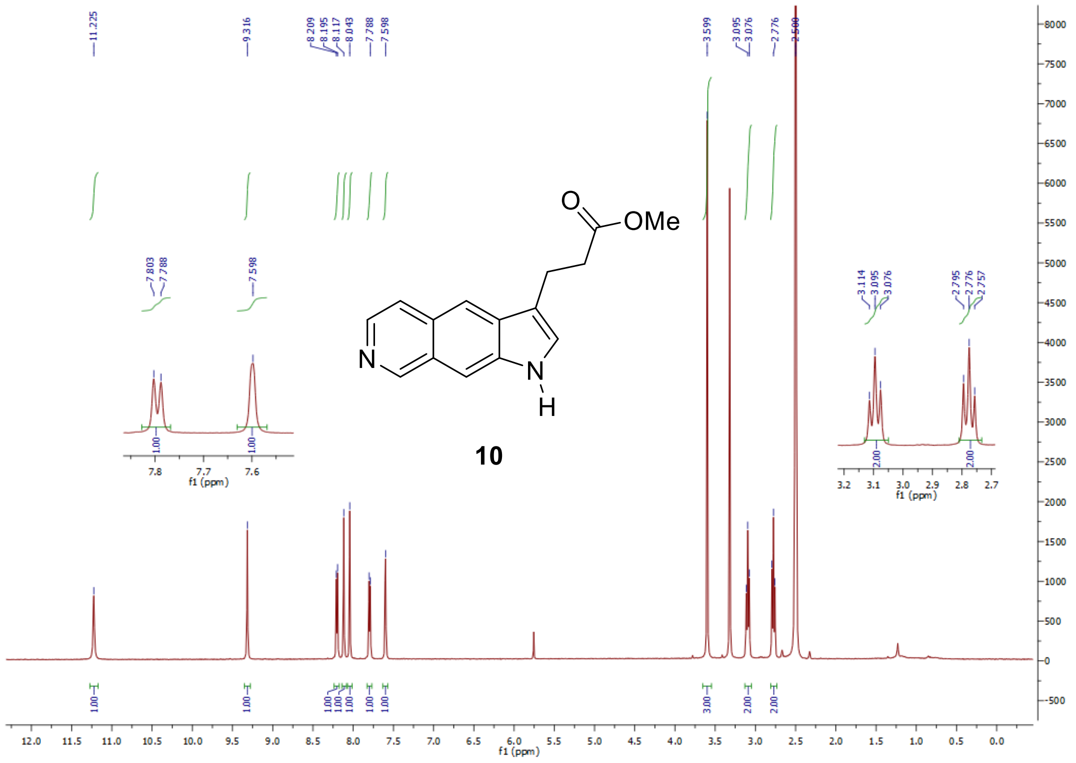

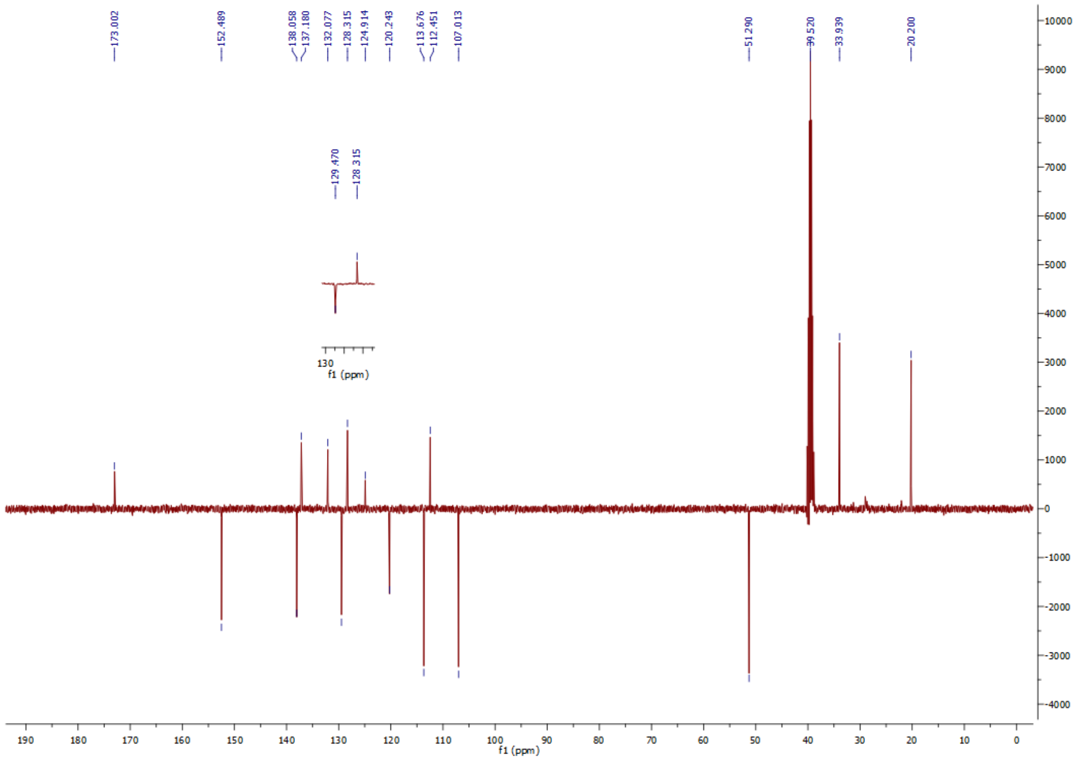

^{1}H NMR and ^{13}C NMR spectra of compound **11**

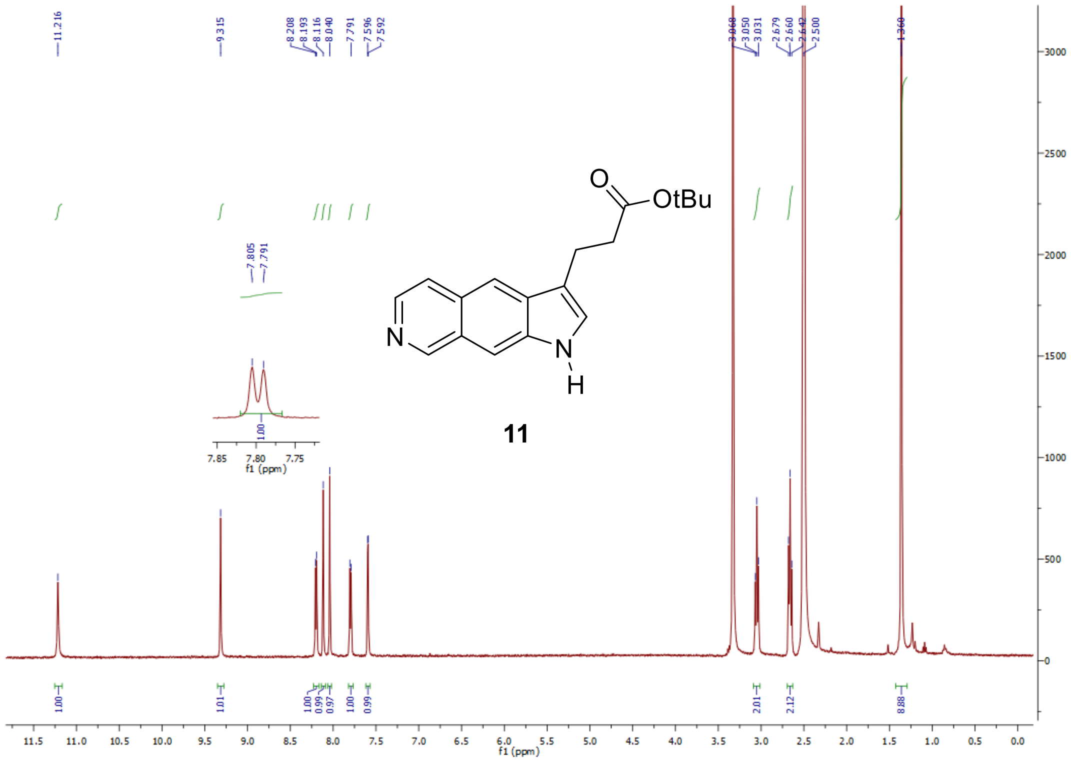

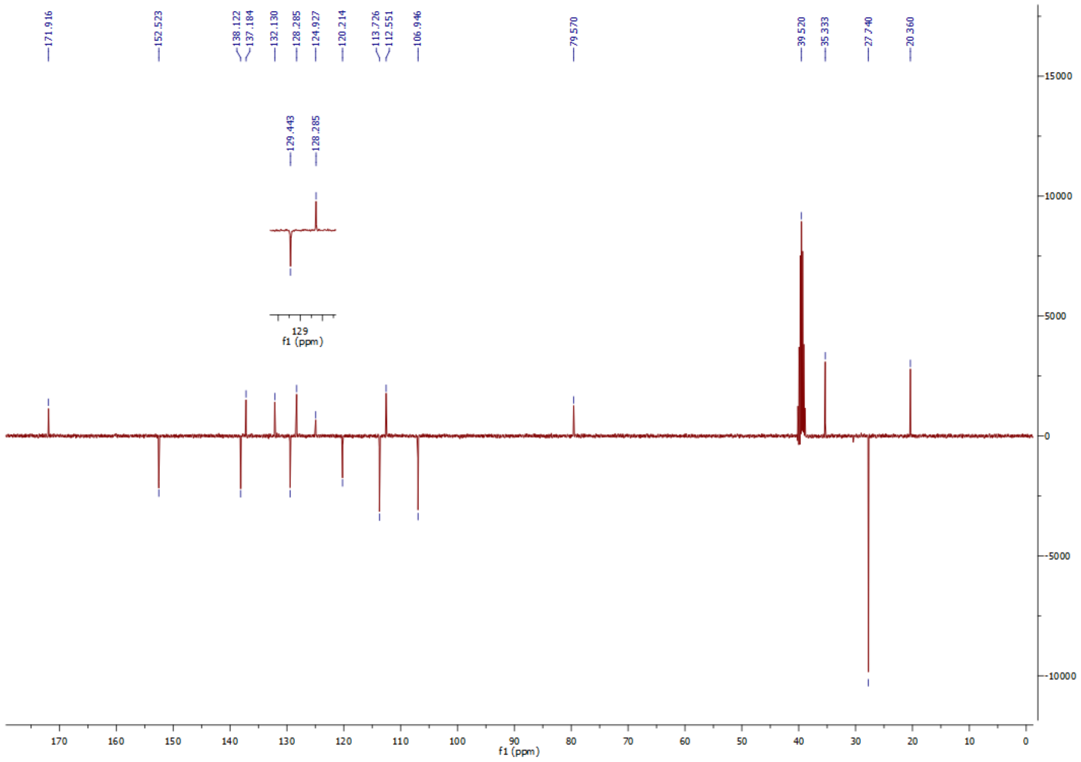

^{1}H NMR and ^{13}C NMR spectra of compound **15**

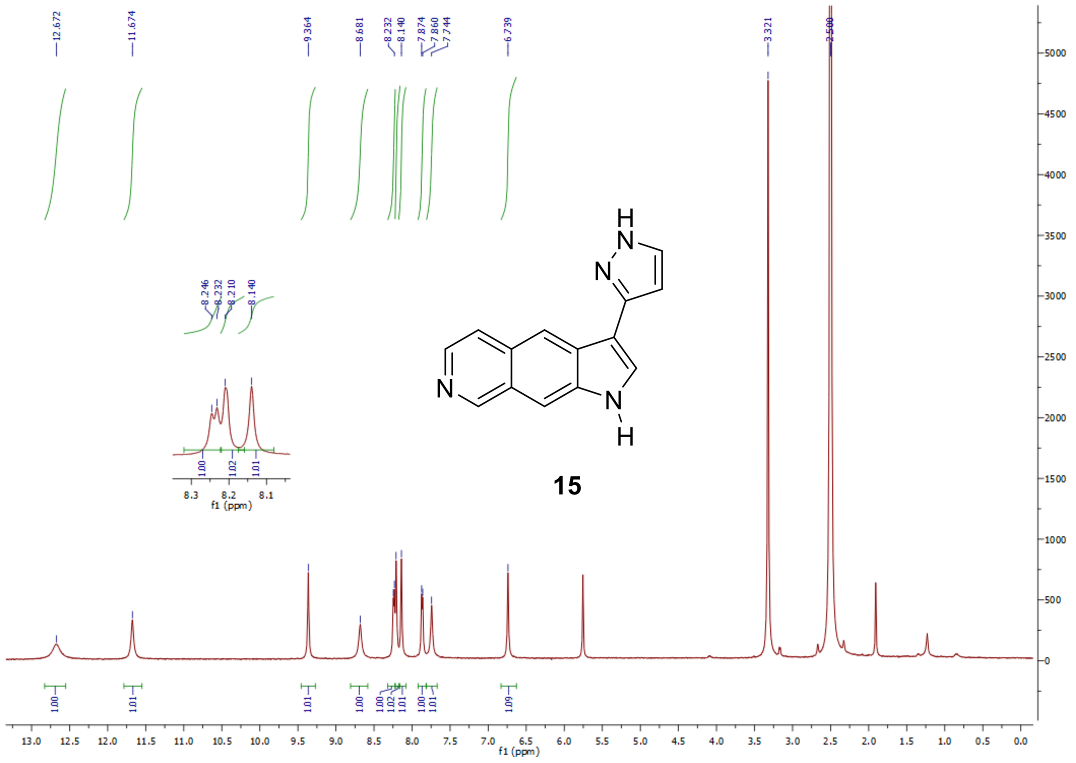

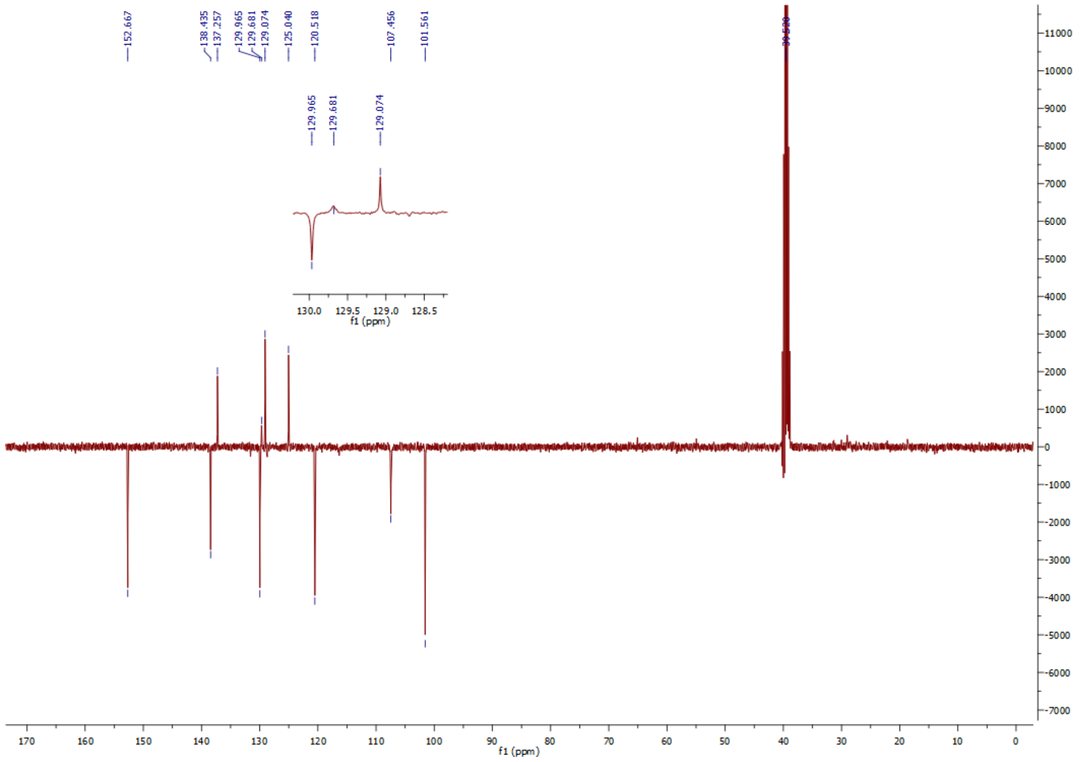

^{1}H NMR and ^{13}C NMR spectra of compound **16**

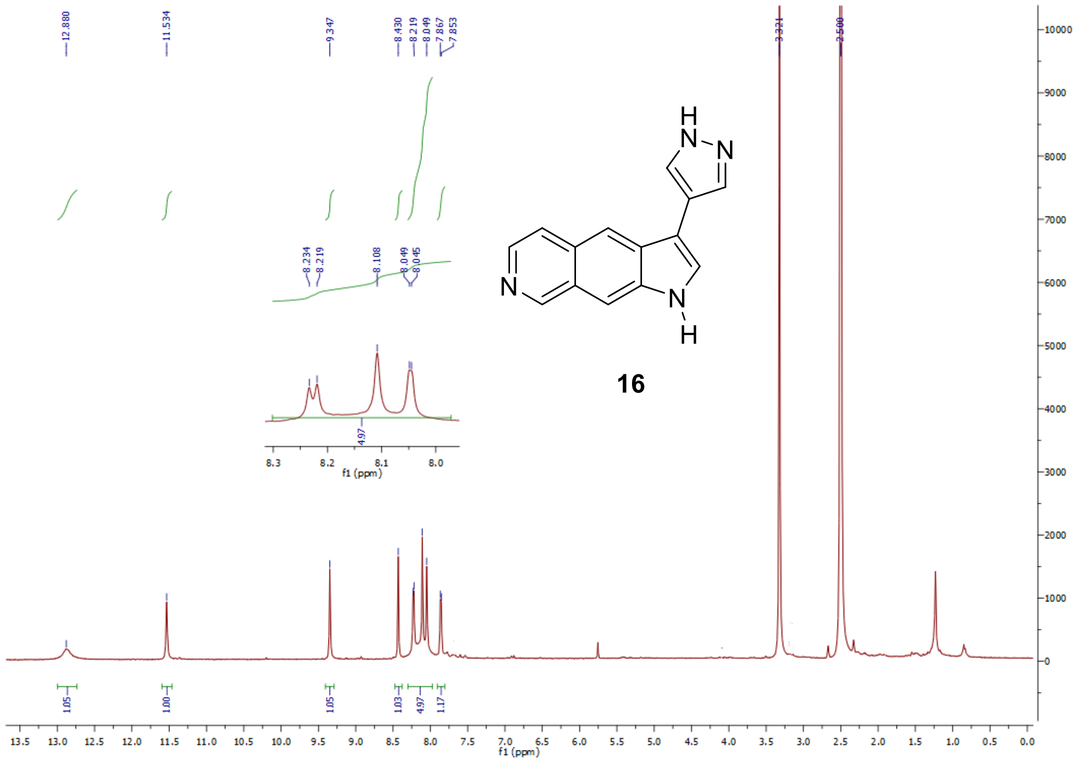

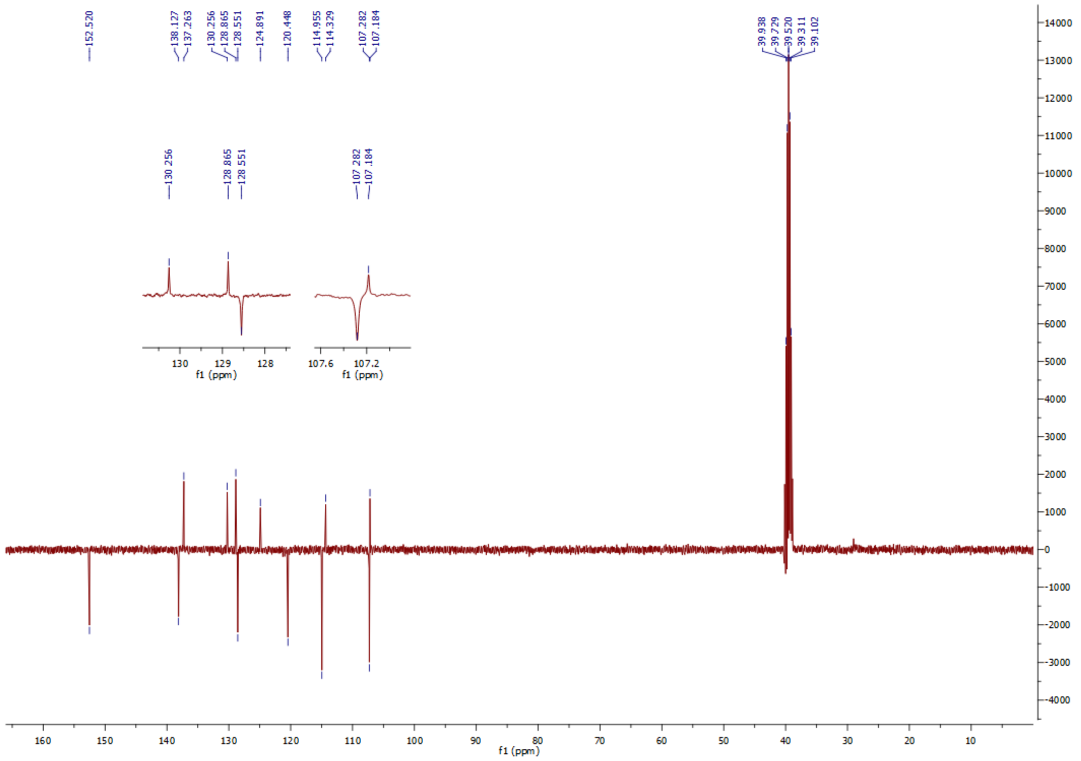

^{1}H NMR and ^{13}C NMR spectra of compound **17**

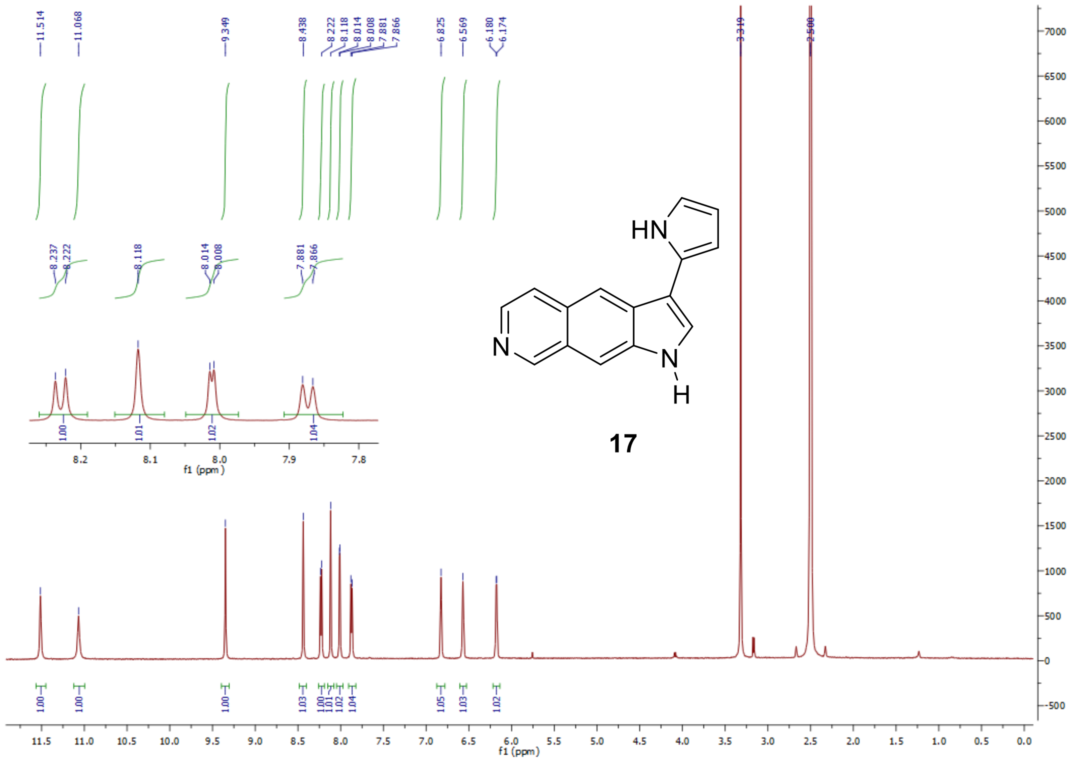

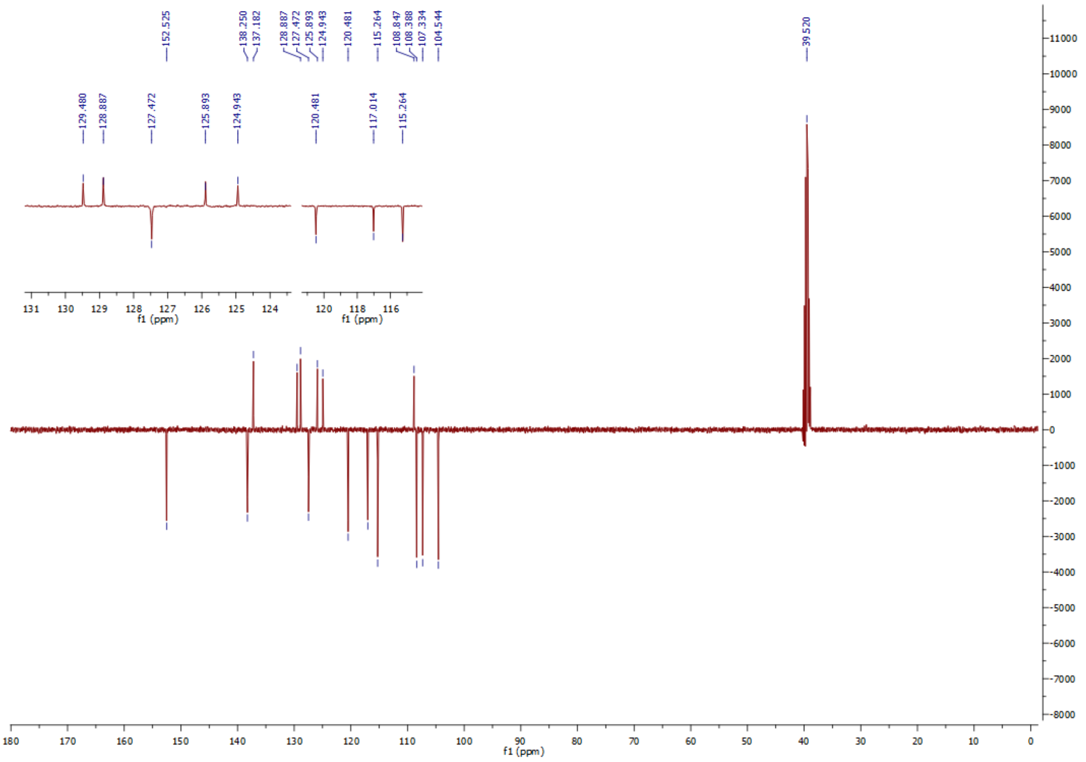

Supplement: Supplementary file 1 [file molecules-30-04388-s001.zip › molecules-3924762-supplementary.pdf]
